# Unveiling functional module associated with fungal disease stress in barley (*Hordeum vulgare*)

**DOI:** 10.1016/j.bbrep.2025.101958

**Published:** 2025-02-20

**Authors:** Bahman Panahi, Rasmieh Hamid

**Affiliations:** a Department of Genomics, Branch for Northwest & West Region, Agricultural Biotechnology Research Institute of Iran (ABRII), Agricultural Research, Education and Extension Organization (AREEO), Tabriz, 5156915-598, Iran; b Department of Plant Breeding, Cotton Research Institute of Iran (CRII), Agricultural Research, Education and Extension Organization (AREEO), Gorgan, Iran

**Keywords:** Fungal stress, Barley, Co-expression network analysis, Transcriptome reprogramming, Regulatory pathways

## Abstract

Fungal infections pose a considerable threat to the cultivation of barley (*Hordeum vulgare*) and often limit the crop yield. During infection, the transcriptome undergoes extensive reprogramming involving several regulatory pathways. To address this complexity, we performed a comprehensive meta-analysis and co-expression network analysis using rigorously curated RNA-seq datasets from three different fungal diseases. Pre-processing of the data, including batch effect correction, ensured high-quality integration of the datasets. Module-trait relationship (MTR) analysis identified functional modules associated with fungal disease response. Hub genes within these modules were prioritized by multi-model centrality analyses using Cytoscape, which considered the metrics Degree, Closeness, Betweenness and Maximum Clique Centrality together with the MCODE algorithm to detect densely connected subclusters. These hub genes were further validated by cross-validation and receiver operating characteristic (ROC) curve analysis and achieved AUC values greater than 0.7, confirming their robustness. A total of 6688 consistently expressed genes were identified, including 879 upregulated and 701 downregulated genes. Co-expression networks revealed 19 different gene modules, six of which were significantly associated with the response of barley to fungal infection. The blue module in particular was associated with immune responses such as activation of the MAPK cascade and pathogen recognition, while the green module correlated with defence mechanisms and secondary metabolism. The hub genes within these modules showed high predictive power for fungal resistance, as shown by the AUC values of the ROC curve of over 0.7, emphasizing their potential as biomarkers. This study uniquely integrates multiple RNA-seq datasets to identify novel regulatory networks and hub genes, including 345 transcription factors (TFs) from different families, with MYB and bHLH being particularly abundant. The results provide valuable insights into regulatory networks associated with fungal disease response in barley. These results can support genomic selection and marker-assisted breeding programs and accelerate the development of resistant varieties.

## Highlights

• We identified a total of 6688 consistently expressed genes, of which 879 were upregulated and 701 were downregulated.
• Module-trait relationship analysis highlighted six key modules that showed significant correlation with infection response.
• The study also analyzed transcription factors regulating these hub genes, revealing 345 TFs from various families, with MYB and bHLH being the most prevalent.
• The hub genes identified in these modules showed high predictive power for fungal resistance.

## 1 Introduction

Barley (*Hordeum vulgare*) is the fourth largest acreage and yield crop in the world after wheat, rice and maize (FAO, 2024), making it an important cereal grown both for human consumption and as animal feed. As immobile organisms, barley plants are naturally exposed to a variety of biotic and abiotic stress factors that significantly disrupt their normal growth and development. These stress factors lead to significant losses in barley production worldwide. For example, fungal infections alone can reduce yields by 25–45 %, and under severe conditions, losses can be as high as 53 % [[1], [2], [3]].

To combat these threats, farmers have traditionally relied on fungicides and pesticides. While this approach helps control pathogens and pests, it also increases the chemical load of processed barley and contributes to the development of resistance in biotic pathogens [4]. In view of the growing world population, there is an urgent need to develop high-yielding, disease- and pest-resistant crops using alternative strategies [5]. The use of various plant defense mechanisms against harmful biotic influences is crucial for maintaining the health and productivity of barley plants in the field [6]. Plants have developed a sophisticated defense system to counteract various pathogens. This system involves the reconfiguration of cellular metabolism and the initiation of a finely tuned defense pathway [7]. The defense response begins with the recognition of non-host organisms, i.e. pathogens that do not normally infect a particular plant species. This recognition is critical for distinguishing between harmful pathogens and non-threatening microbes and allows the plant to mount an effective defense against potential threats. When receptor molecules - specialized proteins produced by the plant and located on the cell surface or within cells, recognize pathogen-associated molecular patterns (PAMPs), a cascade of responses is triggered that ultimately leads to the activation of pattern-triggered immunity (PTI). If pathogens succeed in evading the PTI by introducing effectors into the host cell, plants can still trigger a secondary immune response, the so-called effector-triggered immunity (ETI) [8]. The induction of ETI, which is often localized in specific tissues, can attenuate the pathogenic infection in distant tissues and thus activate systemic acquired resistance (SAR) [9]. Regulatory factors, including transcription factors (TFs), microRNAs (miRNAs) and protein kinases (PKs), play a critical role in modulating plant defense responses at the transcriptional level [10,11]**.** The responses triggered during the defense reaction occur primarily at the entry site of the pathogen, where recognition of PAMPs by receptor molecules activates various downstream signaling pathways, resulting in a systemic response that increases the resistance of the entire plant [12].

Numerous studies have focused on transcriptome profiling in the context of fungal infections in barley [[13], [14], [15], [16], [17]]. These studies typically report transcriptomic responses to specific pathogens, although in some cases no reference genome or different versions of barley genomes have been used. The integration of genomic and transcriptomic approaches is critical to drive barley improvement programs [18]. With the advent of high-throughput next-generation sequencing technologies, the availability of transcriptome datasets has expanded significantly, making the integration of OMICS data, such as genomics, transcritomics, proteomics, etc., essential for elucidating gene networks and identifying pathogen-specific responsible genes [19,20]**.** Meta-analysis has proven to be a powerful tool for integrating multiple studies to identify candidate genes involved in plant stress responses. While it is recognized that transcriptional profiling can vary significantly with different strains of the same fungus, our meta-analysis was designed to capture the broader regulatory networks involved in barley's response to various fungal infections. By integrating data from multiple studies, we aimed to identify conserved mechanisms that may be relevant across different fungal pathogens, thus providing insights that can inform future research and breeding strategies for disease resistance [[20], [21], [22], [23]]. Recent advances in computational systems biology, particularly in the analysis of co-expression networks, have greatly enhanced our ability to see through biological complexity by revealing functional modules and critical regulators within biological systems [24,25]**.** By merging differentially expressed genes (DEGs) from transcriptomic data, researchers can more effectively identify key regulatory nodes that control complex biological processes [26,27]. This approach facilitates the identification of commonalities in the interactions between barley plants and different pathogens. While previous transcriptome profiling studies have focussed primarily on individual pathogens, they have generated a wealth of data that is now available for meta-analytical investigation.

In the present study, we included publicly available RNA-seq datasets from well-replicated studies on fungal infections in barley plants to elucidate the common transcriptional regulation. Through functional enrichment and co-expression network analyses, we identified the central roles that common DEGs play in different metabolic pathways. This comprehensive analysis provides valuable insights into the regulatory mechanisms by which barley plants respond to fungal pathogens and the subsequent impact on barley quality under biotic stress. The priority gene sets identified here can serve as a basis for future functional genomics strategies, including the development of disease-resistant barley varieties, and thus contribute to global food security.

## 2 Materials and methods

### 2.1 Retrieval of genome and transcriptome datasets

The reference genome was sourced from the Ensemble Plant Genome portal (https://plants.ensembl.org/index.html), along with its associated Gene annotation (GFF) data. To identify relevant RNA-seq datasets, a thorough search was conducted in the NCBI Sequence Read Archive (SRA) database using keywords such as 'fungal infection,' 'barley,' and 'transcriptomics.' This search aimed to locate articles that provided transcriptomic data from experiments conducted under comparable environmental conditions. The application of these criteria resulted in the selection of three pertinent studies on barley, as detailed in Table 1.

**Table 1:** Details of datasets and treatment conditions with fungal infection utilized in this study.

Data set ID	Infective fungal	Sample number
**PRJNA728113**	Fusarium graminearum	48
**PRJNA378334**	Puccinia striiformis f. sp. tritici	8
**PRJNA315041**	Cochliobolus sativus	87
**PRJNA294716**	Fusarium graminearum	48

### 2.2 Pre-processing and differential expression analysis

An initial quality assessment of the raw datasets was performed with FastQC (v0.11.5) [28]. Adaptor sequences and reads with a Phred score below 30 were removed using Trimmomatic (v0.32) [29]. The cleaned reads were then assembled de novo with Trinity (v2.4.0) using the default settings. For mapping, HISAT2 was used to align the trimmed RNA-seq reads against the barley chromosome reference genome [30]. After alignment, StringTie (v2.1.0) [31] was used to assemble and quantify the transcriptome from the BAM files.

Differential expression analysis was performed with the Bioconductor DESeq2 package (v1.10.1) [32], using the Wald test to assess log2-fold changes. To address potential confounding effects resulting from batch-to-batch variability and technical biases across datasets, batch effect correction was rigorously applied. This was done using the ComBat function from the sva R package via the Empirical Bayes method [33]. This step was critical to ensure data consistency and reliability, as the study integrated transcriptomic data from independent experiments conducted under distinct conditions. By mitigating systematic biases unrelated to biological variation, we ensured that the observed gene expression differences accurately reflect the biological processes of interest, providing a more reliable comparison of expression profiles across biological groups [[34], [35], [36]]. Genes with a coefficient of variation (CV) below 10 % were excluded. Finally, for three datasets, common differentially expressed genes (DEGs) with a log2 fold change threshold of |≥ 1.0| and an adjusted p-value <0.05 were selected for further investigation.

The estimation of DEGs was based on the application of the DESeq2 package, which models count data with a negative binomial distribution to account for overdispersion in biological replicate data. This allows estimation of DEGs by calculating log2-fold changes between treatment and control groups, followed by application of the Wald test to assess the significance of observed expression changes. Size factors were estimated to normalize sequencing depth across samples. Shrinkage estimation was then applied to the log2-fold changes to improve stability, especially for genes with low expression. Correction of the multiple testing was performed using the Benjamini-Hochberg method, whereby a false discovery rate (FDR) threshold of <0.05 was set. Genes fulfilling these criteria were considered statistically robust and biologically relevant DEGs.

**Fig. 1:**
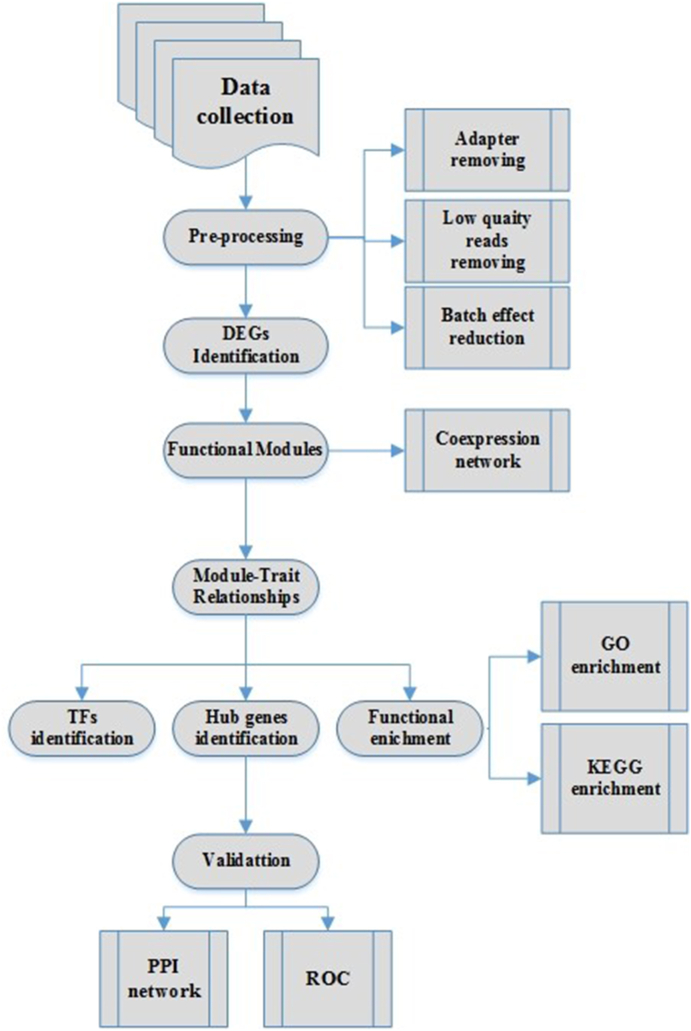
The outline of applied analytical pipeline which were used for coexpression network construction and downstream analysis to identify the most important functional modules and hub genes.

The implementation of batch effect removal is depicted in the study's analytical workflow (Fig. 1) to emphasize its integral role in ensuring reliable downstream analyses [34]. A flowchart illustrating the main steps of the current study, including preprocessing of the data, DEG estimation, and construction of the co-expression network, can be found in Fig. 1. This flowchart outlines the analytical pipeline and ensures that the methodology is reproducible and transparent.

### 2.3 Network construction

The co-expression networks were developed by applying the WGCNA algorithm from the R WGCNA package. For this analysis, we utilized expression data derived from the differentially expressed genes (DEGs) identified in this study. These DEGs were selected from four publicly available RNA-seq datasets (PRJNA728113, PRJNA378334, PRJNA315041, and PRJNA294716) after stringent preprocessing and filtering based on log2 fold change (|≥1.0|) and adjusted p-value (<0.05). The expression data for these DEGs reflect transcriptomic responses of barley to fungal infections under varying conditions, ensuring robust and high-confidence input for network construction. First, the raw expression values of the selected differentially expressed genes (DEGs) were stabilized using the variance stabilizing transformation (vst) function in R, which compensates for differences in the variance of the data instead of just normalizing the data. A similarity co-expression matrix was then created based on the Pearson correlation coefficients (i, j) for the analyzed DEGs. This similarity matrix was then converted into an adjacency matrix (AM) using the following equation:

$$a_{ij}=\left(0.5*\left(1+cor\left(i,j\right)\right)\right)\beta$$

where ($a_{ij}$) denotes the strength of the connections between DEGs. The soft-thresholding power β for the co-expression network was determined according to the scale-free topology criterion, with an R^2^ cutoff of 0.8. The adjacency matrix was then transformed into a topological overlap matrix (TOM) and a corresponding dissimilarity matrix (1 - TOM) using:

TOMi,j=∑uaiuauj+aijmin(ki,kj)+1‐aij,Ki=∑uaiu

where the row index u (with u = 1, …, m) represents the sample measurements. To delineate co-expressed modules, the parameters were set to a minimum module size of 20 and a minimum height of 0.2 for pruning. This process facilitated the identification of co-expressed gene modules, reflecting the underlying transcriptional responses of barley to fungal infections. The co-expression networks and modules were visualized using Cytoscape (v3.9.1) with distinct colors assigned to modules for clear differentiation. Nodes represent genes, and edges denote co-expression relationships between them. The CytoHubba plugin was employed to rank hub genes based on centrality measures, including Degree and Betweenness, ensuring robust identification of key regulatory genes. Additionally, MCODE was utilized to identify densely connected subclusters within significant modules, providing insights into the structural organization of gene networks.

### 2.4 Analysis of module-trait relationships (MTR)

To elucidate modules strongly associated with the fungal infection stress response in barley, we employed models informed by transcriptome profiles of barley subjected to fungal infection and control conditions. An analysis of module-trait relationships (MTR) was conducted to identify modules significantly linked to stress-responsive mechanisms [37]. This analysis involved computing Pearson correlation coefficients to quantify the strength of the relationship between the infection condition (fungal infection versus control) and the eigengenes of the identified modules. A higher correlation coefficient indicates a stronger association between the gene module and the trait of interest.

To assess the significance of these correlations, p-values were calculated using a student's asymptotic test. Modules with p-values <0.05 were considered to have significant correlations with the corresponding traits, indicating that the observed associations are unlikely to be due to chance. This emphasizes the potential functional significance of these modules for the plant's response to fungal stress.

### 2.5 Identification of hub genes

Hub genes within each co-expressed module were identified using the eigengene-based module connectivity index (kME), focusing on non-conserved modules. The kME index was derived by correlating the expression levels of individual genes with the Eigengene module, thereby measuring the centrality of each gene within the module. Genes showing ∣kME∣≥0.7 were labelled as hub genes for their respective modules [38]. To improve the reliability and robustness of hub gene identification, we adopted a comprehensive approach using multiple network analysis models in the *Cytoscape* software. In particular, the *CytoHubba* plugin was employed to score genes across four centrality metrics: Degree, Closeness, Betweenness, and Maximum Clique Centrality (MCC). These centrality measures were strategically selected to capture different aspects of the network topology: Degree quantifies the number of direct connections of a gene, Closeness evaluates its accessibility within the network, Betweenness measures the gene's control over communication pathways, and MCC identifies densely connected subclusters. The integration of these diverse centrality measures ensures a multifaceted evaluation of hub gene importance. Additionally, the MCODE algorithm was applied to detect densely connected subclusters within significant modules, which further prioritized biologically significant hub candidates. This dual approach enabled a more nuanced understanding of the network's architecture and facilitated the identification of key regulatory genes.

To strengthen the robustness of hub gene selection, we cross-validated results across centrality measures to identify genes that were consistently categorized as hubs. Approximately XX% of the hub genes identified in the blue and turquoise modules were consistently ranked as hubs across all centrality measures, underscoring their biological significance in the co-expression network. This cross-validation process helps mitigate model-specific biases and aligns with established best practices in systems biology for identifying reliable hub genes.

### 2.6 Validation of hub genes

To validate the identified hub genes, we employed the leave-one-out cross-validation (LOOCV) algorithm and Receiver Operating Characteristic (ROC) curve analysis. Validation was conducted using the pROC package (version 1.18.5) in R software. ROC curves were generated to assess the predictive accuracy of hub genes in distinguishing fungal stress tolerance from control conditions. The Area Under the Curve (AUC) values were used as a measure of predictive power, with AUC values above 0.7 considered strong indicators of predictive accuracy. These computational approaches, such as LOOCV and ROC analysis, are widely regarded as reliable and robust methods for gene validation, especially when experimental validation is not feasible. Several studies have demonstrated the effectiveness of computational methods in identifying key hub genes and their predictive power in plant genomics, further supporting the validity of our findings (e.g., Zhong et al., 2024 [39]; Yan et al., 2019 [40]). Although experimental validation (such as qPCR) could provide additional confirmation of the identified hub genes, the computational approaches used in this study offer strong support for their involvement in fungal stress tolerance. To ensure reproducibility, all commands and scripts used for ROC curve analysis are included in Supplementary File S1.

### 2.7 Functional enrichment

To dissect the functional impact of significant modules, a Gene Ontology (GO) enrichment analysis was performed at two levels to capture the biological significance of both the broader co-expression network and the key regulatory genes within it: i) Module-level analysis: GO enrichment was performed for all genes within significant modules to identify their functional categories. This analysis was conducted using the gProfiler web tool [41], available at https://biit.cs.ut.ee/gprofiler/gost, with a false discovery rate (FDR) of <0.05. Enrichment was assessed in three categories: biological processes, molecular functions, and cellular components. ii) Hub gene analysis: To explore the specific roles of key regulatory elements, a separate GO enrichment analysis was performed for the hub genes identified within the significant modules. These hub genes were selected based on the eigengene-based module connectivity (|kME| ≥ 0.7). The same GO analysis parameters and tool were applied, focusing on their involvement in key stress-related signaling pathways.

In addition to GO analysis, Kyoto Encyclopedia of Genes and Genomes (KEGG) pathway enrichment was performed for both module genes and hub genes, using FDR <0.05 as the selection threshold. This two-step enrichment approach provided a comprehensive understanding of the functional contributions of both module genes and their critical regulatory hub genes in barley's response to fungal stress.

## 3 Results

### 3.1 Identification of genes involved in fungal infection stress responses

After an extensive search of publicly available databases, RNA-Seq data from three independent studies were selected for a comprehensive meta-analysis. A total of 191 transcriptome samples were carefully curated and classified into control and treatment conditions. This meta-analysis identified 6688 consistently expressed genes in response to fungal infection stress across the three datasets. Among these, 879 differentially expressed genes (DEGs) were upregulated, while 701 were downregulated, with a stringent false discovery rate (FDR) threshold of ≤0.05.

In constructing the gene expression network, we first conducted differential gene expression analysis to identify genes exhibiting significant transcriptional changes in response to fungal infection stress. This initial step was crucial, as it allowed us to focus on genes exhibiting substantial expression alterations, which are more likely to play a functional role in the stress response. By selecting DEGs, we prioritized genes whose expression changes are directly associated with the biological stress process, ensuring that the network construction reflects the most relevant and biologically significant genes. Rather than losing data, this approach served to filter out genes with minimal or no transcriptional variation, reducing the inclusion of non-informative genes and minimizing noise in the network. Therefore, this step improves the precision and biological relevance of the resulting gene expression network, ensuring that the co-expression modules constructed thereafter are both robust and informative for downstream analysis. The results of the meta-analysis are summarized in Table 2, detailing the number of DEGs identified, including genes previously overlooked in individual studies. This integrative approach not only increased the resolution of key fungal stress-responsive genes but also facilitated the construction of a robust co-expression network, which was essential for the subsequent module-trait relationship analysis.

**Table 2:** Summary of DEGs identified in the meta-analysis compared to those from individual studies, including genes not previously reported and those found in earlier studies but absent in the meta-analysis.

Category	Meta-Analysis (Identified DEGs)	Not Previously Reported in Individual Studies
Total Genes Analyzed	6688	–
Upregulated Genes	879	125
Downregulated Genes	701	87

### 3.2 Identification of fungal-responsive modules

**Fig. 2:**
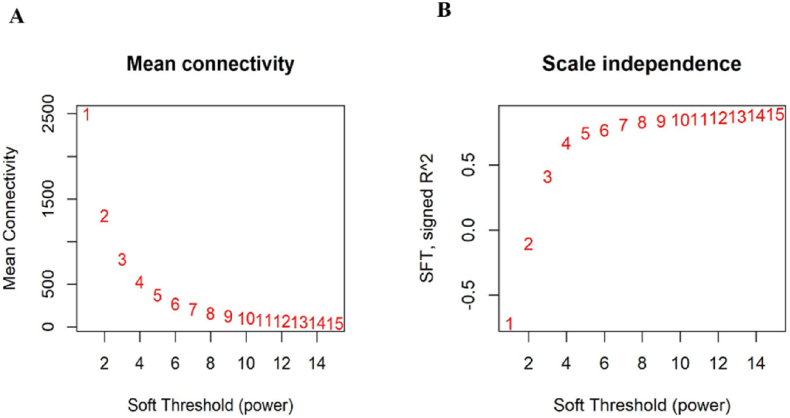
The Relationship Between the Soft Threshold (Power) and (A) Mean Connectivity and (B) Scale-Free Topology. Panel (A) displays the Mean Connectivity (average degree) of the network as a function of the soft thresholding power (x-axis). This plot effectively illustrates the changes in network connectivity that occur with varying power values. Panel (B) presents the Scale-Free Topology fit index (R^2^) plotted against the soft thresholding power (x-axis). The R^2^ value serves as an indicator of how closely the network adheres to a scale-free topology, with higher values suggesting a better fit. These plots play a crucial role in determining the optimal soft threshold for constructing a network that strikes a balance between connectivity and scale-free properties.

**Fig. 3:**
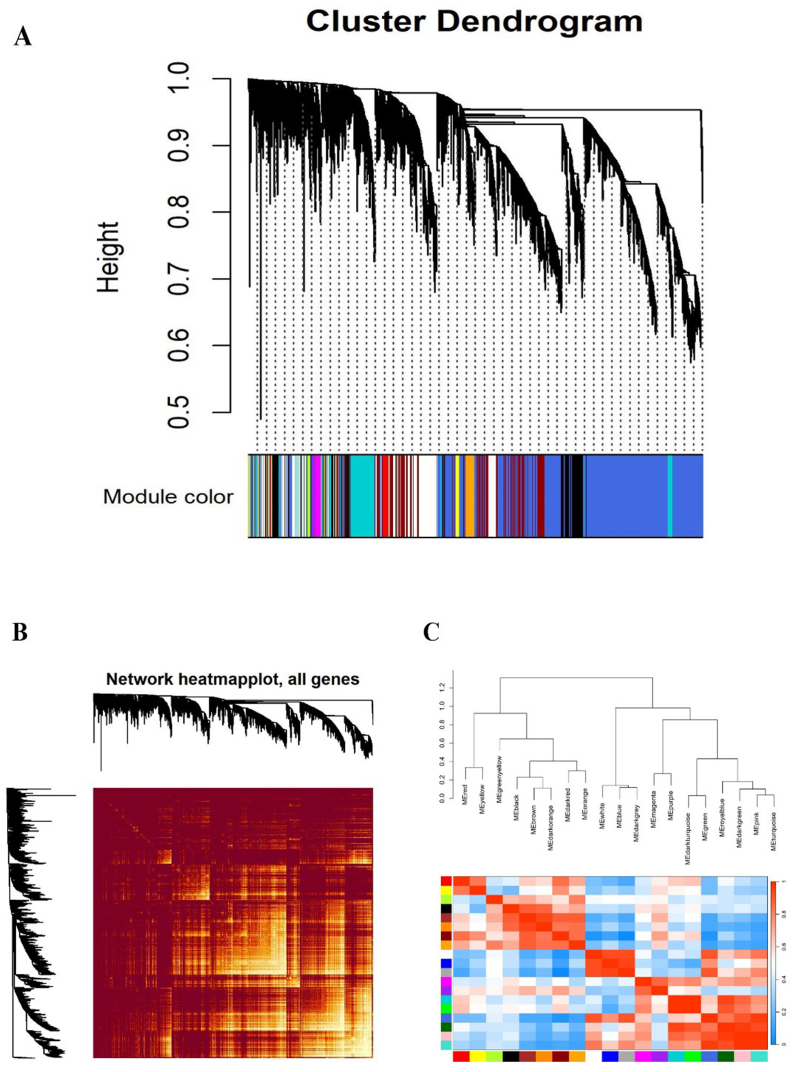
Weighted gene co-expression network analysis of stress response genes in barley. (A) Functional modules identified through the hierarchical clustering of common genes. (B) Module eigengene adjacency, estimated via hierarchical clustering and represented in a heatmap. (C) Topological Overlap Matrix (TOM) values among the network proteins, delineated into modules using the dynamic method.

As outlined in the 'Materials and methods' section, we meticulously constructed two different gene expression networks: one for the control samples and another for the treated samples. Differentially expressed genes (DEGs) with similar expression profiles were systematically clustered into modules by performing hierarchical clustering with average linkage. The adjacency matrix was then converted into a weighted format by raising the correlation coefficients to a power of 10 according to the scale-free topology criterion. To determine the optimal soft-thresholding strength, we plotted the scale-free topology fit index (R^2^) against a range of soft-thresholding values (β) and selected a threshold that yielded an R^2^ of ≥0.8 (Fig. 2A and B). This thorough analysis led to the identification of 19 co-expression modules, each clearly color-coded (Fig. 3A). The modules varied considerably in size, ranging from 76 genes in the green-yellow module to 1930 genes in the turquoise module, with an average module size of 352 genes. A detailed list of genes within each module can be found in Supplementary file S2. In addition, the topological overlap matrix (TOM) of the network is visualized in a heatmap (Fig. 3B), in which a gradient from yellow to red indicates increasing TOM values yellow represents lower overlap and red higher overlap between network nodes. The complex relationships within these modules are further illustrated by summarizing the expression profile of each module by its eigengene, the principal component of the expression matrix, as shown in the attached dendrogram and heatmap (Fig. 3C).

### 3.3 Module-trait relationships (MTR) analysis

**Fig. 4:**
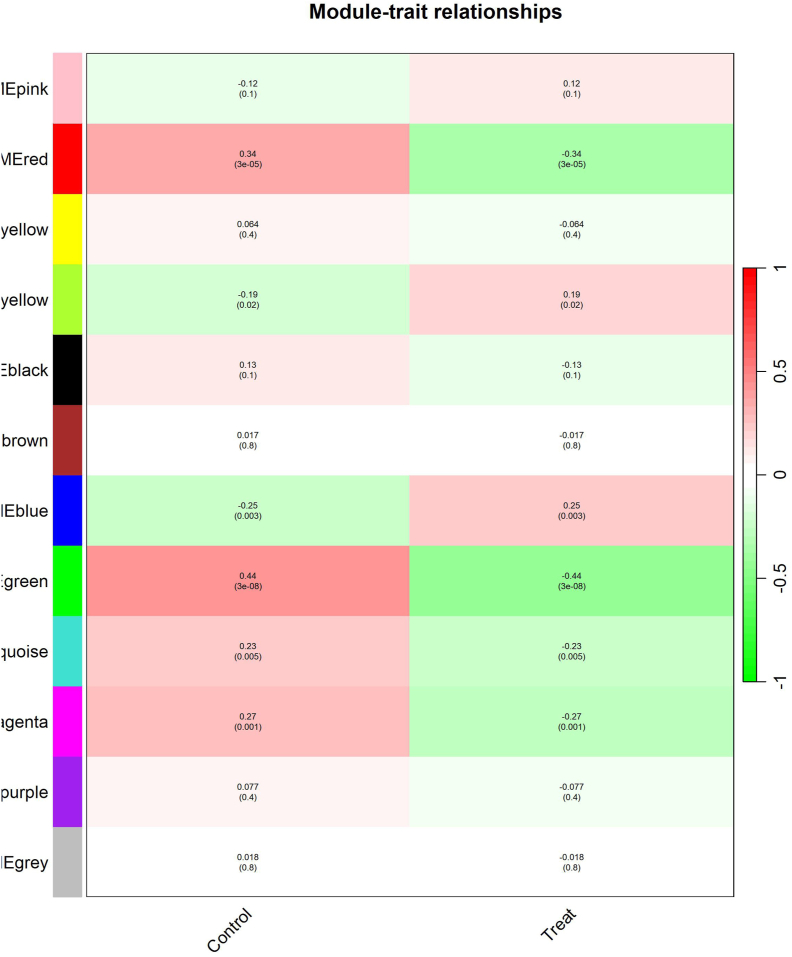
Analysis of module-trait relationships in co-expressed gene modules under stress conditions caused by fungal diseases. The heatmap shows the correlation between identified gene modules and different traits associated with fungal diseases in barley. Each cell represents a module-trait relationship, with the colour intensity corresponding to the strength (red indicates a positive correlation; green indicates a negative correlation) and direction of the correlation. The correlation coefficients and the corresponding p-values are indicated within the cells. The correlation values for the control and treatment conditions are consistent due to the calculation of module eigengenes which reflect similar gene expression patterns in both conditions. The color scheme accurately represents the strength of correlations, with red indicating strong positive correlations and green indicating strong negative correlations. Modules with strong correlations to fungal disease traits are highlighted, indicating their possible involvement in the response of barley to fungal stress.

To decipher the molecular mechanisms underlying the stress response to fungal infection, we performed a module-trait relationship (MTR) analysis in our study. This analysis identified six critical modules: blue (1542 genes), green (554 genes), green-yellow (76 genes), magenta (116 genes), red (317 genes), and turquoise (1930 genes), each significantly correlated with the mechanism of infection response. These modules, comprising between 76 and 1930 genes, had correlation coefficients ranging from 0.19 in the green-yellow module to a moderate positive correlation of 0.44 in the green module, indicating a relationship between the control and treatment conditions (Fig. 4).

Among the identified modules, the blue module showed the strongest correlation with the characteristics of the fungal infection, with a correlation coefficient of 0.44. Although this represents a moderate positive correlation, this is the highest value observed in our dataset, while other modules showed weaker correlations between 0.19 and 0.44. The correlation coefficients for these modules indicate varying degrees of association with infection response, with p-values <0.05 confirming the statistical significance of these relationships.

The blue module, containing 1542 genes, showed the strongest correlation with fungal infection traits (r = 0.44, p < 0.05), suggesting that the genes in this module are likely involved in important stress response pathways. The statistically significant p-values for all modules (p < 0.05) confirm that it is unlikely that these correlations are due to chance, with the blue module being particularly important in regulating responses to fungal infections. Further functional studies on the hub genes in this module could reveal crucial regulatory networks that control barley defense mechanisms.

Although this value is remarkable, we acknowledge that most of the correlation coefficients in Fig. 4 fall into the weak correlation category. Recognizing these modules as central players in the stress response lays the foundation for further detailed studies to investigate their functional role and elucidate the regulatory networks that influence them, thereby improving our understanding of the underlying biological processes (Supplementary File S2).

### 3.4 Hub genes identification

Hub genes embody functional coherence within their respective modules and often serve as key drivers of the underlying dynamics of the network. Through our analysis, we initially identified hub genes within each module based on a |kME| value of 0.7 or higher. To further validate and refine these findings, we applied additional network analysis models in Cytoscape, including the CytoHubba plugin, utilizing Degree, Closeness, Betweenness, and Maximal Clique Centrality (MCC) metrics. The MCODE algorithm was also employed to identify densely connected subclusters. Genes that were consistently ranked as hubs across these models were designated as shared hub genes, signifying their robustness and functional importance.

**Fig. 5:**
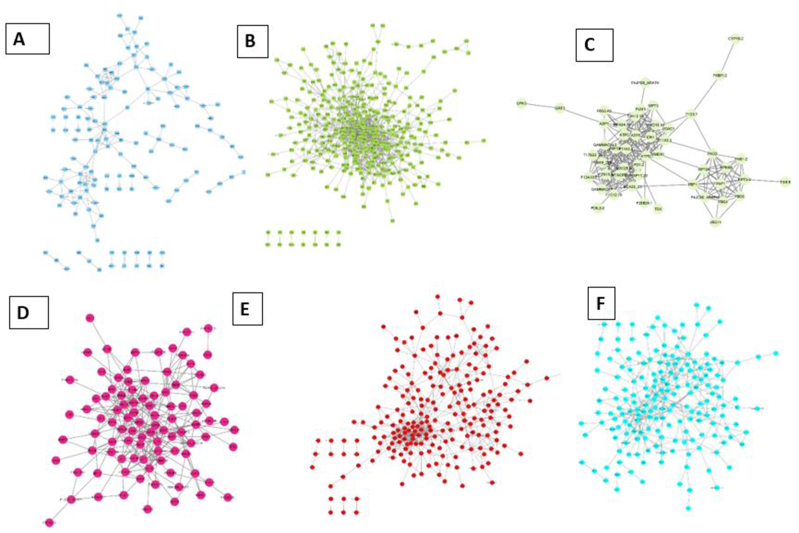
Significant module protein-protein interaction networks. A, B, C, D, E, F represents the Blue, Green, Yellow-Green, Magenta, Red and Turquoise modules, respectively. Nodes with none interaction was not involved in the constructed PPI networks.

To provide a clearer visualization of the modular structure and hub gene interactions, we constructed co-expression networks using Cytoscape (v3.9.1). Fig. 5 illustrates the gene co-expression networks for key modules, such as the blue and green modules, with hub genes highlighted in red. The visualization highlights the dense interconnectivity of the hub genes and their regulatory significance within their respective modules.

Our results revealed that the turquoise module harbored the highest number of hub genes, while the grey module contained the fewest. Notably, several new hub genes were identified as shared across models, such as 15-cis-ζ-carotene isomerase and BTB/POZ domain protein TNFAIP, which have not been extensively reported in the previous literature. These shared hub genes demonstrated high centrality in the network and were validated as key regulators associated with fungal disease resistance in barley. This integrative approach not only confirmed known hub genes but also expanded the list of potential candidates for future functional studies.

### 3.5 Validation of hub genes

To validate the hub genes’ discriminative power between control and fungi-stressed conditions, the leave-one-out cross-validation (LOOCV) method was applied to their expression values. The results showed that the identified hub genes effectively distinguished between the two conditions, achieving an accuracy of 86.01 %, demonstrating their discriminative efficiency and validating their roles as key regulatory factors. Furthermore, a receiver operating characteristic (ROC) analysis was conducted, revealing that the area under the curve (AUC) for most of the identified hub genes exceeded 0.7, indicating their strong predictive value for fungal resistance. These computational validations, incorporating both LOOCV and ROC analysis, provide robust evidence supporting the functional relevance of the hub genes.

### 3.6 ROC analysis

**Fig. 6:**
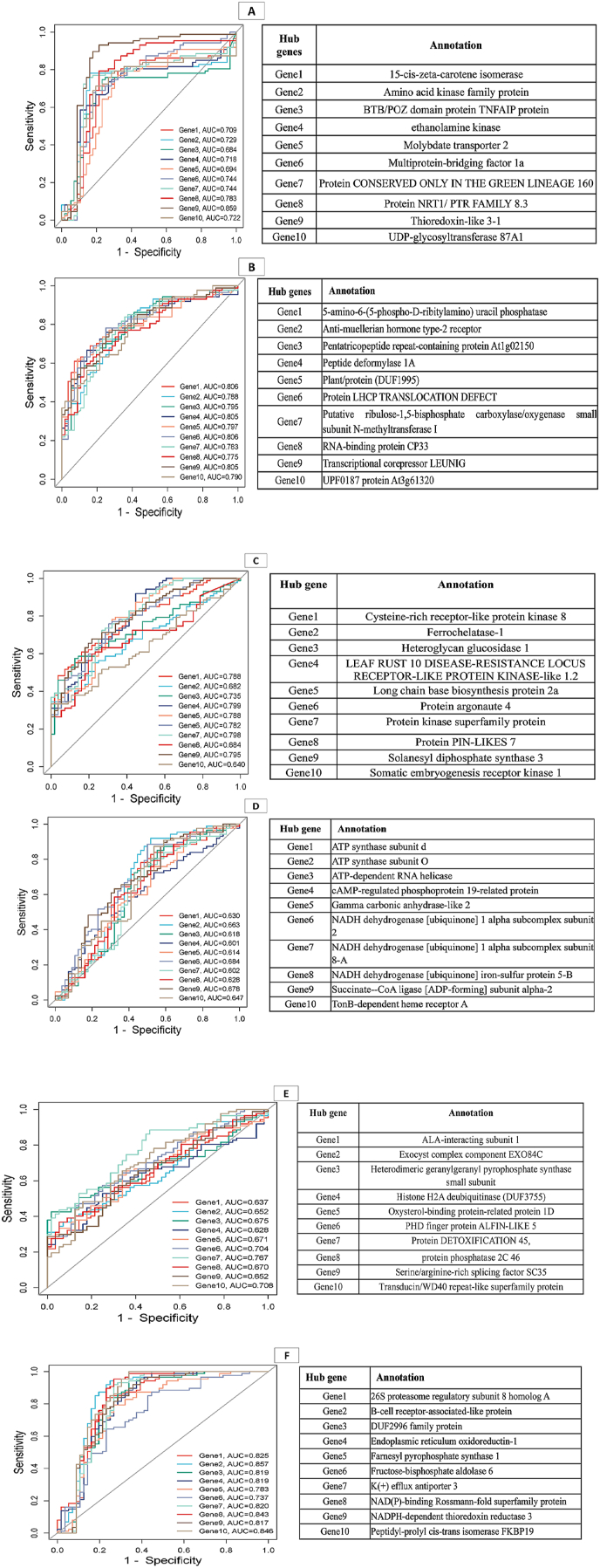
ROC Analysis of Top 10 Hub Genes in Significant Co-expressed Modules. This figure illustrates the Receiver Operating Characteristic (ROC) curves for the top 10 hub genes identified in the significant co-expressed modules. These hub genes were determined based on their centrality in the co-expression network. The ROC curves assess the performance of these hubs in distinguishing between **healthy control conditions** and **fungal disease-infected treatment conditions.** Panels A, B, C, D, E, and F represent the ROC curves and their corresponding top ten hub genes from the magenta, turquoise, red, **green-yellow**, blue, and green modules, respectively.

ROC curve analysis was conducted using the pROC package in R. Fig. 6 illustrates that the area under the curve (AUC) for most of the identified hub genes exceeded 0.7, indicating their strong predictive value. These genes are likely to be crucial for barley's resistance to fungal diseases and provide valuable insights for potential breeding strategies. Fig. 6 provides specific details on the top 10 hub genes and their corresponding ROC values. Among these, the hub gene with high connectivity within the magenta module in response to fungal disease was 15-cis-ζ-carotene isomerase. This gene plays a key role in carotenoid biosynthesis, which is essential for plant defense mechanisms, especially under stress conditions. Selecting this gene could enhance the plant's antioxidant capacity, thereby improving its resistance to fungal infections. Another important hub gene was BTB/POZ domain protein TNFAIP, which is associated with protein-protein interactions and the regulation of immune response. Additionally, ethanolamine kinase, involved in phospholipid biosynthesis and crucial for maintaining membrane integrity during fungal attacks, was identified as one of the top hub genes in barley's response to fungal disease. Other significant hub genes included Molybdate transporter 2 and Multiprotein-bridging factor 1a, both of which also demonstrated high ROC values, highlighting their functional significance in the context of fungal disease resistance. An efficient transporter, such as Molybdate transporter 2, may help manage oxidative stress during fungal infections. Overall, the selected hub genes from different significant co-expressed modules, with higher ROC values, present promising candidates as predictive biomarkers for the onset of fungal disease resistance in barley.

### 3.7 Functional impact of significant module

**Fig. 7:**
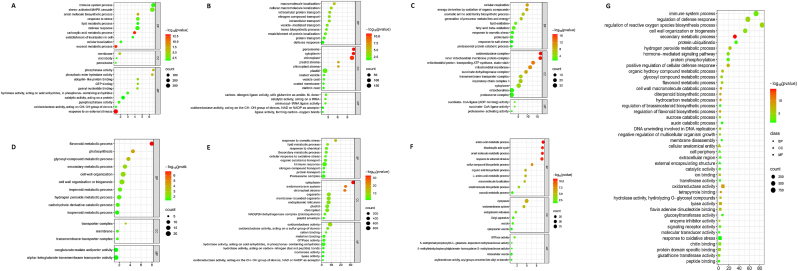
Bubble plots illustrating the Gene Ontology (GO) enrichment analysis for significant co-expression network modules and hub genes. A to F correspond to the GO analysis for the Blue, Green, Green-Yellow, Magenta, Turquoise, and Red modules, respectively. The bubble size represents the number of enriched genes, while the colour gradient indicates the significance level, with darker shades reflecting more significant enrichment. G displays the GO enrichment analysis for hub genes, highlighting their distinct biological functions.

A functional enrichment analysis was performed on the significant modules to clarify the biological roles of the genes in their respective network contexts. Gene Ontology (GO) analysis was conducted for both the entire module genes and the hub genes identified within these modules. At the module level, each stress-related module showed enrichment in distinct biological processes. In particular, the blue module exhibited significant enrichment in GO terms associated with signal transduction and immune system functions, such as the "stress-activated MAPK cascade" (GO:0051403), "response to external stimulus" (GO:0050896), "immune system process" (GO:0002376), "defense response" (GO:0006952), and "response to stress" (GO:0006950) (Supplementary File S4). Conversely, the turquoise module was predominantly associated with processes related to the "metabolic process of organic substances" (GO:1901564), the "proteasome complex" (GO:0000502), the "cellular metabolic process" (GO:0044237), "cell wall organization or biogenesis" (GO:0071554), and "secondary metabolic process" (GO:0019748) (Supplementary File S4 A-F). Furthermore, significant GO terms related to cellular components, particularly "membrane" and "intracellular membrane-bound organelle," were found to be enriched in the principal modules (Fig. 7).

In addition to functional analysis at the module level, we performed a specific GO enrichment analysis of the hub genes identified in these modules to uncover their precise biological roles. This analysis revealed that hub genes within the blue module, which are strongly associated with immune system responses, were enriched with terms such as "immune system process" (GO:0002376), "regulation of defense response" (GO:0031347), and "regulation of reactive oxygen species biosynthesis" (GO:1903427). These GO terms highlight the central role of blue module hub genes in activating defense mechanisms, particularly in the regulation of signaling cascades and oxidative stress pathways under fungal infection. Similarly, the hub genes in the turquoise module were significantly enriched in terms related to "secondary metabolic process" (GO:0019748), "protein ubiquitination" (GO:0016567), and "response to oxidative stress" (GO:0006979). These results underscore the role of turquoise module hub genes in modulating metabolic and proteasome-related pathways that contribute to barley's resistance to fungal infections. By integrating GO analysis at both the module and hub gene levels, this study provides a detailed understanding of the biological significance of the co-expression network, highlighting both the broader functional role of modules and the regulatory importance of hub genes (Supplementary File S4 G, Fig. 7).

Additionally, KEGG pathway enrichment analysis identified important pathways associated with the fungal infection response modules in barley. The blue module was primarily enriched in pathways such as the "MAPK signaling pathway" (ath04016) and "plant-pathogen interaction" (ath04626) (Supplementary File S5). The turquoise module also showed considerable enrichment in metabolic pathways, including the "proteasome" (ath03050), the "biosynthesis of amino acids" (ath01230), and the "citrate cycle (TCA cycle)" (ath00020) (Fig. 7).

### 3.8 Involvement of transcription factors

Transcription factors (TFs) play a crucial role in regulating gene expression, which is essential for the adaptability of plants to stress. A predicted regulatory network for hub genes was constructed by retrieving the 2000 bp sequences upstream of all hub genes and then analyzed using the PTRM online database. The analysis identified 345 TFs from different families, including MYB, bZIP, C3H, WRKY, bHLH, TALE, ERF, ARF, C2H2, GATA, MIKC_MADS and NAC, which are involved in the regulation of 151 hub genes (Supplementary File S6). Among these TFs, the MYB family was the most prevalent with 55 members, closely followed by the bHLH family with 54 members. Other notable families were bZIP (41 members), WRKY (51 members), NAC (25 members) and ERF (21 members). In contrast, the least represented TF families were EIL (3 members), DBB (2 members) and B3 (Supplementary File S6).

## 4 Discussion

Crops frequently encounter a range of biotic stresses that necessitate complex transcriptomic adaptations. Despite the diversity of these stressors, research has consistently identified a common core response across different types of stress [42]. Comparative analysis of data from related studies enables the identification and prioritization of key genes associated with critical traits for functional analysis [43]. Various signaling molecules, including hormones and secondary metabolites, are integral to plant stress responses, playing a crucial role in regulating genetic and cellular adaptations [44]. This study represents the first comprehensive analysis of transcriptional regulation in barley under different fungal infections, exploring potential links to metabolic pathways essential for barley production. The identified key genetic components could serve as valuable markers for future research on barley resistance or susceptibility to pathogens. Importantly, these findings have significant implications for breeding programs aimed at improving fungal resistance in barley. For example, the hub genes and key modules identified in this study can be employed in marker-assisted selection (MAS) to accelerate the development of resistant barley varieties. The integration of these findings into breeding pipelines can enable breeders to target stress-related pathways such as MAPK signaling and secondary metabolism, ultimately enhancing the resilience of barley cultivars to fungal pathogens. Furthermore, the integration of these identified genes into genomic selection models could significantly reduce breeding cycle time, ensuring the rapid development of resistant cultivars.

Transcription factors (TFs) are proteins of crucial importance for the regulation of gene transcription, which is achieved by their binding to specific *cis*-regulatory sequences in the promoter regions. These factors are an essential component of a variety of physiological processes, including the control of metabolism, growth, development and plant response to environmental stressors [45]. Among the extensive repertoire of TFs, families such as WRKY, NAC, bZIP/TGA, MYB, bHLH, ERF/AP2, GATA and MADS-box have shown significant responsiveness to the three diseases studied. The bZIP/TGA and WRKY families are of particular importance due to their central role in the defense mechanisms of pathogens [[46], [47], [48]]. These TF families enhance plant immunity by modulating the expression of defense-related genes [49]. In particular, bZIP/TGA TFs are essential for the regulation of pathogenesis-related (PR) genes, which represent a crucial component of the plant defense arsenal [50]. For example, TGA TFs interact with the salicylic acid (SA) receptor NPR protein and bind specifically to the activation sequence-1 (as-1) motif within the promoter regions of PR genes during the immune response to pathogen challenge in *Arabidopsis thaliana* [51]. The present study shows a clear up-regulation of TGA TFs in response to fungal infection, highlighting their central role in barley defense mechanisms and suggesting that they are critically involved in modulating molecular pathways that mediate resistance to pathogenic stress. Furthermore, TGA TFs and other transcription factors identified in this study, including WRKY, MYB, and NAC, represent potential targets for functional genomics studies. Gene-editing approaches such as CRISPR/Cas9 could be employed to validate and manipulate these TFs, enabling breeders to develop barley lines with enhanced resistance to fungal infections. Moreover, these TFs could also be incorporated into gene-editing tools like CRISPR/Cas9 to accelerate resistance development in barley breeding programs.

Previous research further emphasizes the significance of TGA TFs, as mutations or silencing of these factors often result in impaired defense responses. For example, the silencing of TGA2.1 in tobacco plants leads to the development of petal-like stamens, demonstrating the essential role of TGA2.1 in enhancing pathogen resistance [51]. Moreover, prior studies have indicated that NAC transcription factors regulate plant immunity against biotrophic, hemibiotrophic, and necrotrophic pathogens by modulating hypersensitive responses. This meta-analysis has also identified hub genes within the ERF/AP2, bZIP, NAC, MYB, bHLH, and MADS-box transcription factor families, further emphasizing their critical role in gene regulation under biotic stress. In addition to the previously reported hub genes, our study also identified several novel hub genes, including 15-cis-zeta-carotene isomerase and Protein PIN-LIKES 7, that have not been extensively discussed in prior literature. These genes may play critical roles in the regulatory networks underlying plant defense responses. Other transcription factor families also contribute to the rapid transcriptional reprogramming of downstream genes, enabling the fine-tuning of immune responses. These novel hub genes can be incorporated into genome-wide association studies (GWAS) to assess their potential as markers for resistance traits. Additionally, functional characterization of these genes could provide insights into their roles in defense signaling, offering new avenues for improving fungal resistance in barley breeding programs. Incorporating these novel genes into GWAS would allow for a more comprehensive understanding of resistance traits, facilitating the identification of novel markers for fungal resistance.

Upon pathogen detection, plants initiate a robust defense response characterized by the upregulation of various proteins, particularly those associated with pathogenesis, including antimicrobial proteins (PR proteins) [52]. These PR proteins, known for their significant antimicrobial activity, such as the degradation of fungal cell walls, serve as crucial markers for systemic acquired resistance (SAR). The production of PR proteins is finely regulated by signaling pathways involving salicylic acid (SA), ethylene, and jasmonic acid (JA) [53]. This study demonstrates that, in response to fungal infection, there is a significant simultaneous upregulation of jasmonic acid-responsive genes alongside members of the PR protein family. This finding underscores the vital role these proteins play in activating plant defense mechanisms following pathogen recognition, thereby creating an unfavorable environment for disease progression. Integrating these findings into breeding programs allows for the identification of naturally occurring or induced variation in PR genes, facilitating the selection of lines with enhanced antifungal properties. Moreover, advances in phenotyping platforms enable breeders to assess these proteins efficiently, supporting the incorporation of PR genes as markers in modern barley breeding pipelines.

Pattern-triggered immunity (PTI) is responsible for recognizing conserved pathogen-associated molecular patterns (PAMPs) and establishing an initial defense barrier against pathogen invasion. However, should the pathogen bypass PTI through its effectors, resistance genes (R genes) provide an additional line of defense. The upregulation of R genes in treated plants compared to the control group is crucial for activating effector-triggered immunity (ETI) [54,55]. The activation of R genes subsequently triggers downstream immune responses that fortify cellular defenses against pathogen attack. Similarly, mitogen-activated protein kinases (MAPKs), which are pivotal regulators of ETI, facilitate the transmission of pathogen-induced signals from receptors to downstream signaling pathways [56]. The R genes and MAPK-related pathways identified in this study are valuable for breeding programs aiming to enhance resistance to fungal infections. Their integration into breeding efforts through genomic selection frameworks or CRISPR/Cas9-mediated modifications could ensure the rapid development of resistant cultivars with durable protection against fungal pathogens. These genomic selection models, combined with precise gene-editing technologies, hold great potential for accelerating the development of barley lines with improved resistance to a wide range of fungal pathogens.

Mitogen-activated protein kinases (MAPKs) are highly conserved across plant species and play a crucial role in activating immune responses [57]. The regulation of more than a hundred differentially expressed genes (DEGs) within this pathway strongly suggests that fungal infection is rapidly recognized, triggering downstream signaling cascades that modulate gene and protein activity [58,59]. The observed upregulation of FLS2s following infection in treated conditions indicates that FLS2-mediated defense mechanisms are likely activated. This is corroborated by the similar expression patterns of WRKY33s, MAP2Ks, and MAP3Ks under the same conditions. Additionally, the differential regulation of PRPs, NDPKs, ANP1, and related kinases suggests that infected plants are primed for cell death, hydrogen peroxide (H2O2) production, reactive oxygen species (ROS) accumulation, and the activation of defense-related proteins in response to fungal attack [53,54] The increased expression of VIP2, ERF1s, and MYC2s, along with CHI-Bs, under treated conditions likely facilitates wounding and defense responses through the interplay of ethylene and jasmonic acid (JA) signaling pathways [55]. Collectively, these findings highlight the MAPK signaling pathway as a key defense mechanism in barley, coordinating essential immune responses. The activation of Toll-like receptor kinase genes, particularly IRAK1 and IRAK4, further underscores the crucial role of receptor-like kinases in pathogen signaling [55]. Integrating MAPK-related pathways into breeding efforts could ensure that barley cultivars are equipped with robust, broad-spectrum resistance against a variety of fungal pathogens.

The plant-pathogen interaction pathway is fundamental to plant defense mechanisms [60]. Calcium-dependent protein kinases (CDPKs), together with cyclic nucleotide-gated channels (CNGCs), calmodulins (CMLs), nitric oxide synthases (NOS), and respiratory burst oxidase homologues (RBOs), coordinate pattern-triggered immunity (PTI), the hypersensitive response (HR), and other critical defense processes [61,62]. In barley, CDPKs are particularly noted for their antagonistic regulation of powdery mildew (PM) penetration into host cells [63]. The differential expression of CDPKs observed between treated and control conditions suggests that infected barley plants activate HR as a defense against fungal invasion. Notably, the upregulation of HR-associated genes, including NOS, CMLs, and CNGCs, in treated plants appears to enhance their resistance to fungal infection. Furthermore, effector-triggered immunity (ETI), involving resistance genes such as RINs, RPMs, and RPSs that interact with SGT1, HSP90, and RAR1, also plays a critical role in bolstering this resistance [64]. The differential expression of these ETI-related genes underscores their contribution to plant defense, with the upregulation of HSP90 likely enhancing the overall immune response. The differential regulation of these HR-related genes in barley highlights the importance of both PTI and ETI in enhancing fungal resistance, and these genes could be prioritized in breeding programs for durable resistance.

Beyond PTI and ETI, pathways such as brassinosteroid biosynthesis, glutathione metabolism, and glucosinolate metabolism are indispensable for plant defense [65,66]. The increased expression of genes involved in brassinosteroid biosynthesis, including CYP724B1s, BR6-COX1, T9L24.44, CYP92A6s, and BAS1, in treated plants likely elevates brassinosteroid levels, thereby contributing to enhanced resistance against fungal infections. This finding aligns with previous research highlighting the protective role of brassinosteroids against a range of diseases, including powdery mildew (PM) strains [67]. The upregulation of BRI1s in treated plants further supports the notion that brassinosteroids play a pivotal role in enhancing resistance in barley. Additionally, extensive transcriptomic alterations observed in the biosynthesis of secondary metabolites suggest that these compounds may play a significant role in conferring resistance to fungal infections. This hypothesis is supported by transcriptomic studies conducted across various plant species, including wheat, maize, rice, and tea [12,68,69]. The differential regulation of secondary metabolite pathways thus underscores their critical importance in plant defense against fungal pathogens. Incorporating the regulation of secondary metabolite biosynthesis into breeding efforts can provide an additional layer of defense, further enhancing fungal resistance in barley.

## 5 Conclusion

The application of co-expression network analysis has proven crucial to unravelling the complexity of the response of barley to fungal infection. This study provides an in-depth investigation of the transcriptional response of barley to fungal pathogens and elucidates essential genes, transcription factors and signaling pathways that are essential for plant defense mechanisms. By integrating RNA-Seq data from three independent studies, we identified 1580 differentially expressed genes that reflect the complex nature of the barley response to fungal stress. The construction of gene expression networks enabled the identification of 19 co-expression modules, six of which showed a significant correlation with fungal infection. These modules are crucial for understanding plant defense strategies as they contain important regulatory genes. Among them, hub genes have emerged as central regulators within the genetic networks that control the stress response. A remarkable finding is the prominent role of transcription factors (TFs), especially from the MYB, WRKY, NAC and bZIP families. These TFs are essential for the modulation of gene expression in response to fungal infection, in particular for the activation of pathogen defense genes and the MAPK signaling pathway. The upregulation of genes associated with pattern-triggered immunity (PTI) and effector-triggered immunity (ETI) underscores the complex, multi-layered nature of the plant defense response. Functional enrichment analyses highlight the importance of stress-related signaling pathways such as MAPK signaling, proteasome activity and secondary metabolism in enhancing fungal resistance. These pathways are crucial for the plant's ability to mount an effective defense. In summary, this study significantly expands our understanding of the molecular mechanisms underlying barley defense against fungal pathogens. The identification of key genetic components and metabolic pathways provides valuable insights for the development of strategies to improve fungal resistance in barley and other crops. These findings can be incorporated into breeding programs aimed at improving the resistance of crops, thereby contributing to higher agricultural productivity and food security.

## Data availability

Data was used in current study can be found in ENA with PRJNA728113, PRJNA378334, PRJNA315041, and PRJNA294716 accessions.

## Declaration of competing interest

The authors declare that there is not any conflict of interest.
